# 2225. Analysis of Co-Resistance Among *Escherichia coli* Urine Isolates From Female Outpatients in the United States

**DOI:** 10.1093/ofid/ofac492.1844

**Published:** 2022-12-15

**Authors:** Keith S Kaye, Vikas Gupta, Aruni Mulgirigama, Ashish V Joshi, Nicole E Scangarella-Oman, Kalvin Yu, Janet Watts, Fanny S Mitrani-Gold

**Affiliations:** Rutgers - Robert Wood Johnson Medical School, New Brunswick, New Jersey; Becton, Dickinson and Company (BD), Franklin Lakes, New Jersey; GlaxoSmithKline plc., Surrey, England, United Kingdom; GlaxoSmithKline plc., Surrey, England, United Kingdom; GlaxoSmithKline plc., Surrey, England, United Kingdom; Becton, Dickinson and Company (BD), Franklin Lakes, New Jersey; Becton, Dickinson and Company (BD), Franklin Lakes, New Jersey; GlaxoSmithKline plc., Surrey, England, United Kingdom

## Abstract

**Background:**

In the past 10 years, there has been a substantial increase in antimicrobial resistance among uropathogens in community-acquired uncomplicated urinary tract infections (uUTIs), including extended spectrum β-lactamase producing (ESBL+) Enterobacterales and multidrug resistance. This study examined urine isolates from female outpatients in the United States (US) for co-resistance among *Escherichia coli* (*E. coli*).

**Methods:**

This was a retrospective, cross-sectional study of 30-day non-duplicate *E. coli* urine isolates (first isolates collected within a 30-day period) from female outpatients (≥ 12 years of age) at 304 US facilities. Included patients had ≥ 3 months of data from 2011 to 2019 (Becton, Dickinson and Company [BD] Insights Research Database). Urine-isolated *E. coli* were defined as ESBL+ by 1) commercial panel or 2) not susceptible (NS; intermediate/resistant) to ceftriaxone, cefotaxime, ceftazidime, or cefepime), or NS to any of: fluoroquinolones (FQs), trimethoprim/sulfamethoxazole (SXT), or nitrofurantoin (NFT). Microbiological co-resistance phenotypes were characterized in isolates NS to ≥ 2 of the 4 resistance phenotypes assessed.

**Results:**

In total, 856,918 unique isolates were evaluated. Co-resistance data are shown in the Table. Of ESBL+ isolates (96,306), 72.4% were co-resistant to FQ, 56.7% to SXT, and 11.9% to NFT; 6.8% had all 4 phenotypes. For FQ NS isolates (319,354), 21.8% were also ESBL+, 51.6% were co-resistant to SXT, 8.0% were co-resistant to NFT, and 2.0% had all 4 phenotypes. Among SXT NS isolates (384,304), 14.2% were also ESBL+, 42.9% were co-resistant to FQ, 6.8% were co-resistant to NFT, and 1.7% had all 4 phenotypes. Finally, for NFT NS isolates (56,954), 20.1% were ESBL+, 44.7% were co-resistant to FQ, 46.0% were co-resistant to SXT, and 11.5% had all 4 phenotypes.

**Table.**
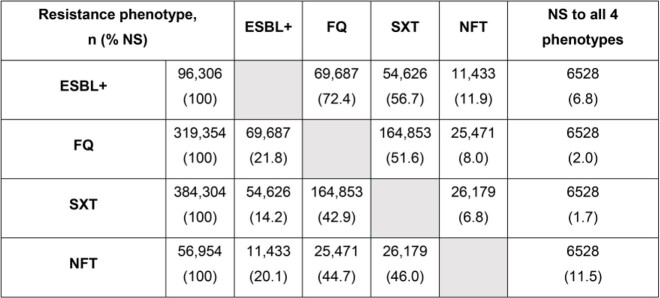
Co-resistance phenotype combinations among urine-isolated Escherichia coli (2011–2019)

**Conclusion:**

Among urine-isolated *E. coli*, there was a high prevalence of co-resistance, particularly for ESBL+ isolates where co-resistance to FQ was > 70%. The availability of effective oral treatments for uUTI is limited by uropathogen antibiotic resistance. These data may help inform appropriate empiric prescribing practices to optimize the treatment of uUTI, mitigate multiple drug exposure for a uUTI event, and potentially influence long-term resistance patterns among *E. coli*.

**Disclosures:**

**Keith S. Kaye, MD, MPH**, Allecra: Advisor/Consultant|GlaxoSmithKline plc.: Receiving symposia honoraria|GlaxoSmithKline plc.: GlaxoSmithKline plc.-sponsored study 212502|Merck: Advisor/Consultant|qpex: Advisor/Consultant|Shionogi: Grant/Research Support|Spero: Advisor/Consultant **Vikas Gupta, PharmD**, Becton, Dickinson and Company: Employee of, and shareholder in, Becton, Dickinson and Company, and the company received funding from GlaxoSmithKline plc. to conduct this study|GlaxoSmithKline plc.: GlaxoSmithKline plc.-sponsored study 212502 **Aruni Mulgirigama, MBBS**, GlaxoSmithKline plc.: Employee and shareholder|GlaxoSmithKline plc.: GlaxoSmithKline plc.-sponsored study 212502 **Ashish V. Joshi, PhD**, GlaxoSmithKline plc.: Employee and shareholder|GlaxoSmithKline plc.: GlaxoSmithKline plc.-sponsored study **Nicole E. Scangarella-Oman, MS**, GlaxoSmithKline plc.: Employee and shareholder **Kalvin Yu, MD, FIDSA**, Becton, Dickinson and Company: Employee of, and shareholder in, Becton, Dickinson and Company, and the company received funding from GlaxoSmithKline plc. to conduct this study|GlaxoSmithKline plc.: GlaxoSmithKline plc.-sponsored study 212502 **Janet Watts, PhD**, Becton, Dickinson and Company: Employee of Becton, Dickinson and Company, and the company received funding from GlaxoSmithKline plc. to conduct this study|GlaxoSmithKline plc.: GlaxoSmithKline plc.-sponsored study 212502 **Fanny S. Mitrani-Gold, MPH**, GlaxoSmithKline plc.: Employee and shareholder|GlaxoSmithKline plc.: GlaxoSmithKline plc.-sponsored study 212502.

**Table.** Co-resistance phenotype combinations among urine-isolated Escherichia coli (2011–2019)

In total, 856,918 non-duplicate (30-day) Escherichia coli isolates were evaluated.

Note: Some isolates had overlapping susceptibilities/antimicrobial resistance types, hence individual phenotype totals do not reflect total number of isolates evaluated.

Abbreviations: ESBL+, extended spectrum β-lactamase-producing (by commercial panel or not susceptible to ceftriaxone, cefotaxime, ceftazidime or cefepime); FQ, fluoroquinolone; NFT, nitrofurantoin; NS, not susceptible; SXT, trimethoprim/sulfamethoxazole.

